# A novel collision tumour of myofibroblastoma and low-grade adenosquamous carcinoma in breast

**DOI:** 10.1186/s13000-020-00992-2

**Published:** 2020-06-23

**Authors:** Guang-Zhi Yang, Shang-Hua Liang, Xiao-Hong Shi

**Affiliations:** Department of Pathology, Beijing Dian Medical Test Laboratory Co. Ltd, No. 9 Tianfu Street, Daxing District, 102600 Beijing, People’s Republic of China

**Keywords:** Collision tumour, Myofibroblastoma, Low-grade adenosquamous carcinoma, Breast

## Abstract

**Background:**

Myofibroblastoma (MFB) and low-grade adenosquamous carcinoma (LGASC) are rare tumours in the breast, respectively. However, a collision tumour of the two types has never been reported.

**Case presentation:**

A 42-year-old female presented with a palpable mass in diameter of about 2.5 cm in the left breast. Morphologically, the lesion was predominately composed of bland spindle cells admixed with some islands of mature adipocytes and a few epithelial elements dispersing in infiltrating way which formed both tubule and solid structures. The mass showed low positive index of Ki-67. The spindle cells were strongly and diffusely positive for CD34, SMA, desmin, ER and PR. The epithelial elements were positive for CK and EMA, and negative for ER and PR completely. CK5/6 and P63 were positive in the outer-layer of the tubules and nearly all the cells of the solid nests.

**Conclusions:**

A collision tumour of MFB and LGASC in breast is extremely rare and either component is supposed to be not overlooked. Excision and close follow-up are advised.

## Background

Myofibroblastoma (MFB) of the breast is an uncommon stromal tumour with a variety of myofibroblastic differentiation just as the name shows [1]. In recent years, many unusual morphologic variants besides the classical type have been recognized [2]. Few cases of MFB associated with another lesion have been reported [3].

Low-grade adenosquamous carcinoma (LGASC) is a low-grade variant of metaplastic carcinomas (MC) with favorable prognosis in the breast [4]. LGASC has been seldom reported to be associated with other neoplasms such as adenomyoepithelioma (AME) [5].

We report an extremely rare collision tumour of MFB and LGASC in the breast. In our knowledge, this is the first case and it will expand the spectrum of the both neoplasms.

## Case presentation

A 42-year-old female presented with a palpable mass in the upper outer quadrant of her left breast. Physical examination revealed no evidence of nipple discharge and remarkable changes in the right breast and both axillary areas. The patient received no radiographic examinations and pre-operative needle biopsy. The mass was resected at the local hospital and fixed in 10% neutral-buffered formalin for examination.

**Fig. 1:**
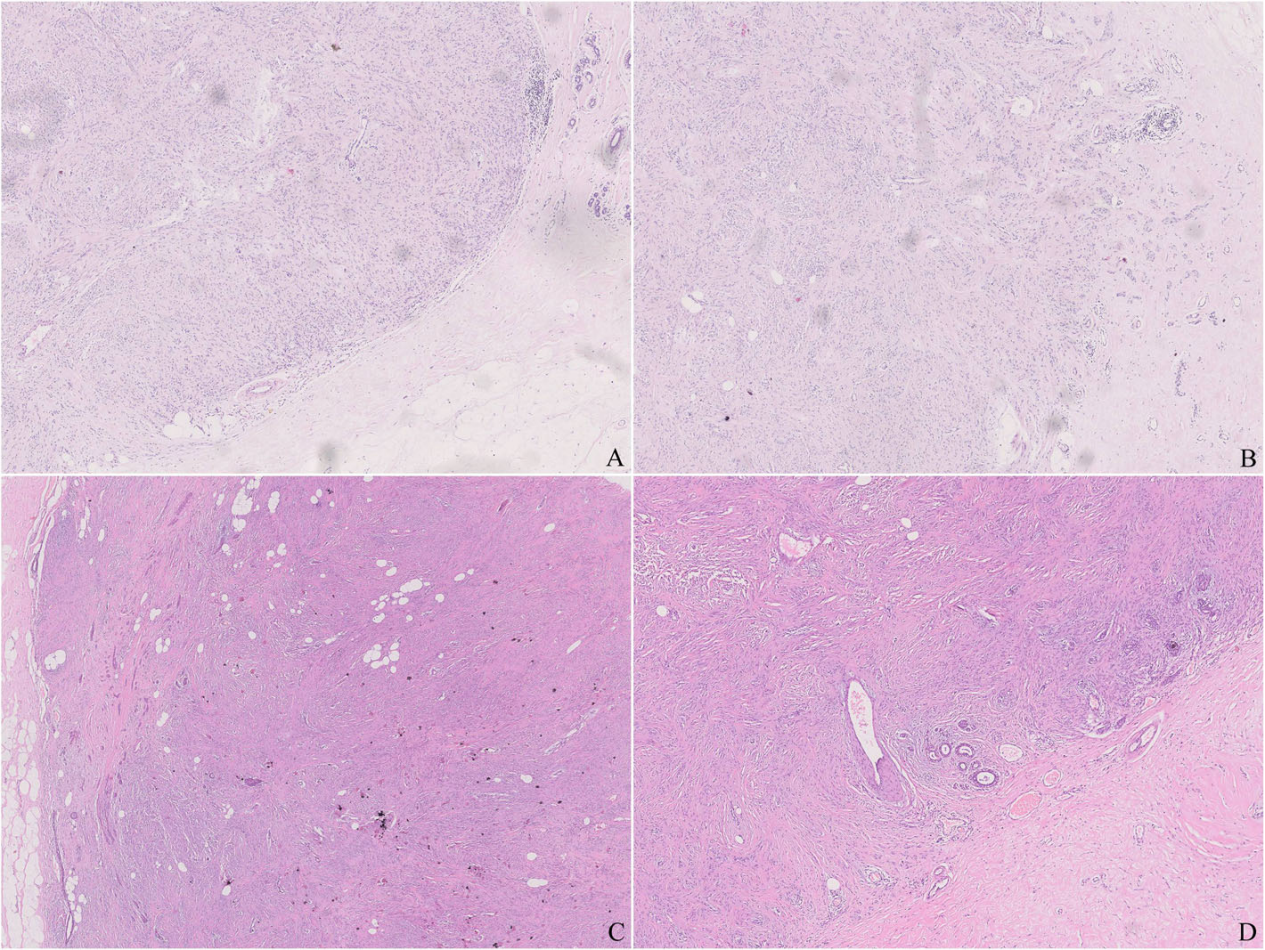
**a**. The mass without envelope was well circumscribed. **b**. The mass was infiltrating in foci. **c**. Some epithelia were involved in the mass as a component of the mass. **d**. Some mammary glands with lobular outline were entrapped as a proof for infiltration

**Fig. 2:**
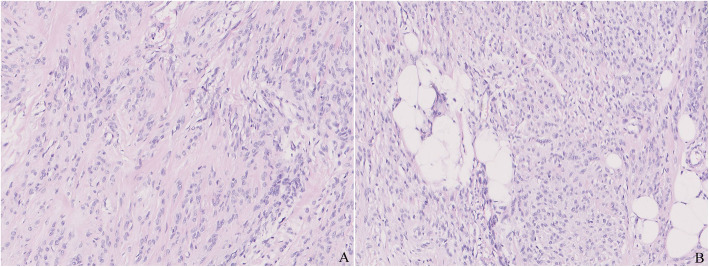
**a**. The mass was predominately composed of bland spindle cells with absence of mitoses and necrosis. **b**. A few islands of mature adipocytes admixed in the mass

**Fig. 3:**
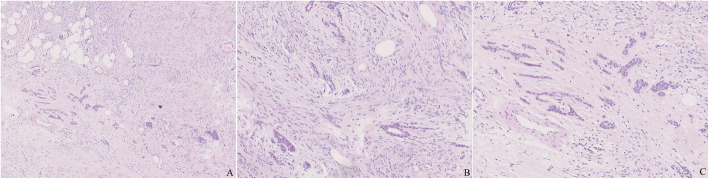
**a**. There were a few bland epithelial elements with formation of tubule or solid structures in a haphazardly infiltrating manner. **b**. The tubules, composed of two-layer cells, resembled normal mammary glands. **c**. The solid nests were composed of uniform cells without distinct squamous metaplasia

The lesion was about 2.5 cm in diameter with a thin layer of normal mammary lobules and mesenchyme around and thus appeared completely although narrowly excised. The mass was relatively well circumscribed without envelope in most areas, while infiltrating the vicinity focally (Fig. 1). The lesion predominately consisted of dense proliferation of spindle cells with abundant eosinophilic cytoplasm and a spindle nucleus with inconspicuous nucleolus inside. Although arranged densely, they were bland-looking in cytological morphology, and neither mitosis nor necrosis was observed. Some islands of mature adipocytes dispersed in the mass (Fig. 2). Except the spindle cells and adipocytes, there were a few bland epithelial elements in the peripheral area of the mass, which presented in a patch accounting for up to 3% of the entire tumour (Fig. 3). They formed both tubule and solid structures without hemorrhage, degeneration, inflammation or calcification nearby. The tubules were composed of two-layer cells and the solid cells were uniform without distinct squamous metaplasia. They appeared one component of the neoplasm rather than the surrounding mammary glands entrapped in the mass due to distorted outline in variable degree and absence of lobular structure. Actually, they dispersed in a haphazardly infiltrating manner without desmoplasia.

**Fig. 4:**
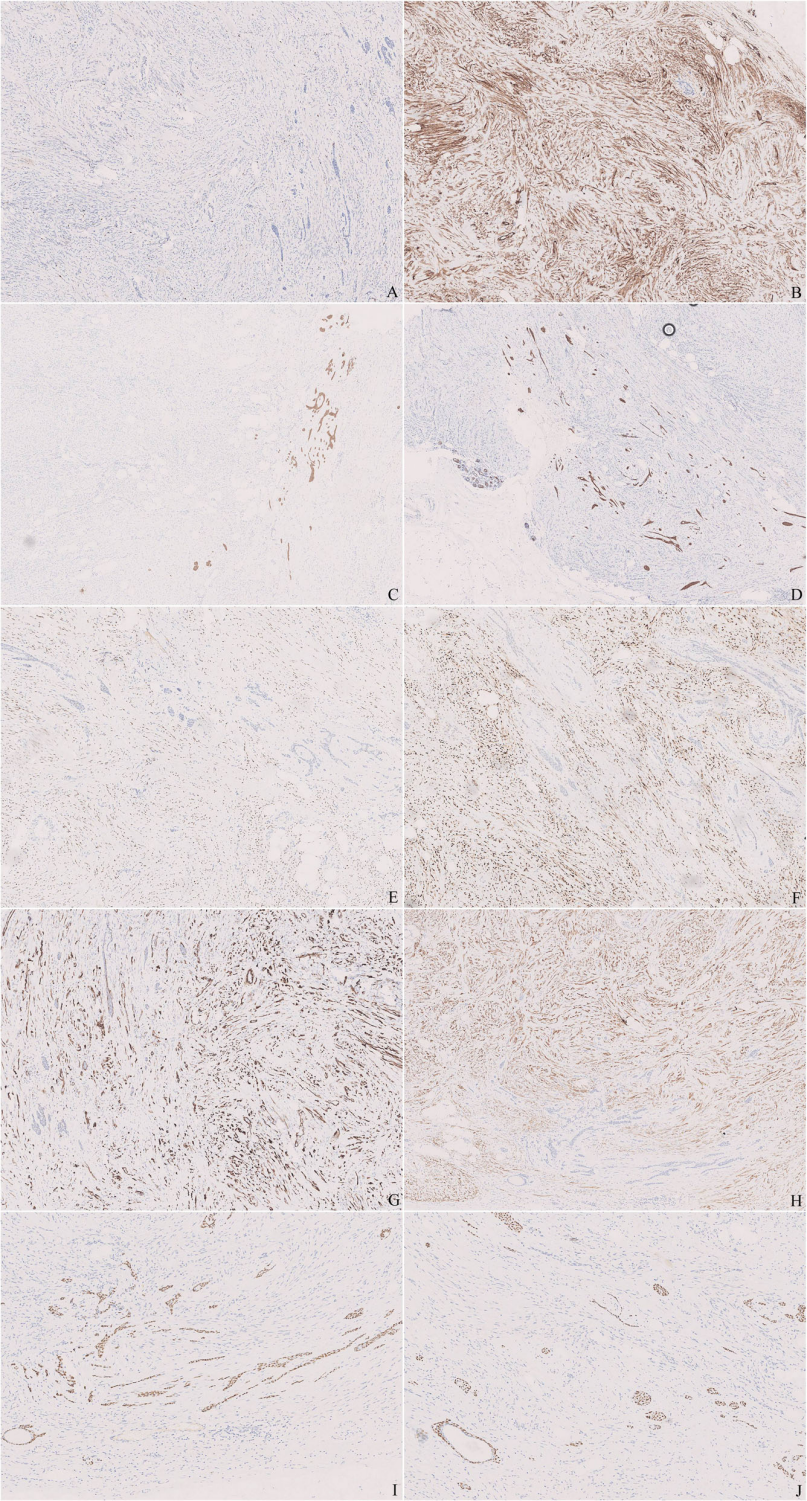
Immunohistochemistry. **a**. Ki-67 showed very low proliferating index. **b**. CD34 was strongly and diffusely positive in the spindle cells. **c** and **d**. CK and CK5/6 were negative in the spindle cells but strongly positive in the epithelial elements, which confirmed the characteristics and displayed the distorted outlines. **e** and **f**. ER and PR were diffusely positive in the spindle cells and negative in the epithelial elements. **g** and **h**. SMA and desmin were diffusely positive in the spindle cells, which suggested myogenic differentiation. SMA was positive patchily and desmin was negative in the outer-layer of epithelial elements. **i** and **j**. P63 was positive in the outer-layer of the tubules just as that in the normal glands, and in nearly all the cells of the solid nests

Immunohistochemistry was also performed with antibodies of pan-cytokeratin (CK), epithelial membrane antigen (EMA), CK5/6, estrogen receptor (ER), progesterone receptor (PR), P63, smooth muscle actin (SMA), Calponin, CD10, desmin, CD34, bcl-2, Ki-67 (Fig. 4). Both spindle and epithelial cells demonstrated low positive index of Ki-67. The spindle cells were strongly and diffusely positive for CD34, SMA, desmin, ER and PR and negative for other markers. Positivity of CK and EMA confirmed the characteristics of the epithelial elements. CK5/6 and P63 were positive in nearly all the cells of the solid nests, but only in the outer-layer of the tubules just as the pattern of myoepithelia around the normal glands. SMA, CD10 and Calponin were positive patchily, which provided little information, and other markers were negative.

Integrated by morphology and immunostaining, the spindle cells were diagnosed as MFB and the epithelial elements as LGASC. Thus, collision tumour of MFB and LGASC was the permanent pathology. Since either of the collision is benign or low-grade malignant, respectively, and the total mass was excised, no further treatment was adopted. Follow-up was recommended with timely re-excision for any potential recurrence. So far, there has been no recurrence for one-year follow-up.

## Discussion and conclusions

Spindle cell lesions, exclusively or predominately composed of spindle cells, are frequently encountered in breast pathology. Diagnosis of high-grade spindle cell lesions is seldom problematic. However, low-grade lesions are among the most difficult to diagnose due to bland spindle cells and absence of necrosis or mitosis. Low-grade spindle cell lesions were present in the case. Nodular fasciitis, fibromatosis, myoepithelioma (ME), pseudohemangiomatoid stromal hyperplasia (PASH), solitary fibrous tumour (SFT), MFB, benign or borderline phyllodes tumour (PT), low-grade sarcoma, some variants of MCs, and so on, are all listed for differential diagnosis.

As the basic pathological diagnostic rule of breast spindle cell lesions, MC must be considered first because the incidence of other types of lesions even if added together is lower than that of MC. In the case, the diagnosis of fibromatosis-like MC was insufficient as none of pan-CK, CK5/6 and EMA was positive in the spindle cells. Another compelling evidence was that ER and PR were diffusely positive because MC has been generally thought to be hormone receptors negative [6].

Since there were a few epithelial cells in the spindle cell crowds, diagnosis of fibroepithelial neoplasm seemed to be desirable if these two components were regarded as the components of a tumour. PT was reasonably considered due to over-growth of mesenchyme, lack of atypia or mitosis and focal invasion of around normal tissue. However, the perfect morphological exploration was challenged by immunohistochemistry. Although the immunophenotype is uncertain and variable, it is known that spindle cells of PT are hormone receptors negative [7].

Actually diagnoses were quite limited due to diffuse expression of ER and PR. Almost all above-mentioned low-grade spindle cell lesions are ER and PR negative except MFB [8]. Both morphological features and expression of other immunostaining markers, including SMA, desmin and CD34, supported the diagnosis of MFB.

However, the epithelia entrapped within the mass remained confusing since it was rare in MFB. The epithelia were morphologically bland and P63 was positive around some tubules, leading to the impression of normal and benign glands. It is known that some displaced epithelial cells appearing normal or atypical may present along the biopsy track. The lesion hadn’t been biopsied and was absent from hemorrhage, degeneration, inflammation or calcification, which usually are valuable for diagnosis of displacement. Thus, the epithelia were supposed of an intrinsic part rather than displacement.

So far, a case of MFB was reported as originated from the mammary hamartoma with hypocellular area composed of mammary glandular tissue in a lobular arrangement, fibrous stroma, and adipose tissue in variable proportions [9]. This probability was excluded in this case as such a background didn’t exist. The alternative was that the epithelial cells were evidence of mass invasion. In fact, a rare infiltrating subtype has been recognized despite that most MFBs are non-encapsulated with pushing borders [10]. In addition, it should be emphasized that the case was different from epithelioid-cell MFB, which is a rare morphologic variant exclusively or predominantly composed of cells with epithelioid morphology [2]. The neoplastic cells exhibited variable degree of nuclear pleomorphism and wide variety of growth patterns, including alveolar, linear, and solid patterns. However, the epithelioid cells are verified of myofibroblastic cells rather than real epithelial cells by immunohistochemistry.

The above analyses were based on the deceitful and questionable hypothesis that the epithelia were normal or benign. The tubules were somewhat distorted in outlines, lacked lobular structure and distributed in the infiltrating way. Although the solid epithelial nests didn’t display distinct squamous metaplasia morphologically, they did express P63 and CK5/6 strongly and diffusely, which are always regarded as markers of squamous cells. It was also worth noting that ER and PR were completely negative while normal mammary glands are positive in variable proportions. Thus the diagnosis of LGASC was established.

The terminology of collision and composite tumours needs to be re-visited in the case. The occurrence of two or more synchronous tumours in the same organ is supposed to be named for collision tumours, in which the components are separated from each other without histological admixture. Those of the composite tumours are so intimately admixed with each other as to be separated topographically impossibly, and are regarded as divergent lineages originating from the same neoplastic clonal proliferation. Although MFB and LGASC in the case were admixed, they should be rather named as a collision tumour due to no proof for the same origin. To our knowledge, a collision tumour of MFB and LGASC in the breast has never been reported until now.

Our report expanded the spectrum of MFB and LGASC, respectively. There are only a few reports about accompany. It was reported that MFB was associated with spindle cell lipoma (SCL) with expression of S-100 in foci [3]. It may be argued because MFB and SCL had similar chromosomal aberrations and there was a genetic link between them [11]. As far as LGASC is concerned, it was reported to be associated with AME as they merged in some area [5]. Although LGASC is widely regarded as one kind of carcinomas, it suggested that LGASC may arise from myoepithelia of AME [5]. In our case, the two components of epithelial cells and spindle cells appeared not related to each other, yet the origin of LGASC and MFB is to be elucidated.

Prognosis and treatment of a collision tumour vary greatly. It is understood that prognosis is mainly determined by the more aggressive tumour. Obviously, treatment is a more complicated dilemma, especially when both of the components are malignant but treatments share little. Fortunately, it was not a problem in our case. MFB is benign and LGASC is low-grade malignant, and both follow indolent courses with a greater predilection towards local recurrence than distant metastasis, typically managed with lumpectomy and follow-up. In our case, the lump was excised and no recurrence or metastasis was seen after one-year follow-up.

In conclusion, we reported an extremely rare case of novel collision tumour of MFB and LGASC in breast. Either component should not be overlooked. Surgical resection and close follow-up are advised. The etiology, origin and relationship remain to be fully elucidated.

## Data Availability

The dataset supporting the conclusion of this article is included within the article.

## Abbreviations

MFB: Myofibroblastoma
LGASC: Low-grade adenosquamous carcinoma
MC: Metaplastic carcinoma
AME: Adenomyoepithelioma
CK: Cytokeratin
EMA: Epithelial membrane antigen
ER: Estrogen receptor
PR: Progesterone receptor
SMA: Smooth muscle actin
ME: Myoepithelioma
PASH: Pseudohemangiomatoid Stromal Hyperplasia
SFT: Solitary fibrous tumour
PT: Phyllodes tumour
SCL: Spindle cell lipoma
